# Recent Research Progress on Polyamidoamine-Engineered Hydrogels for Biomedical Applications

**DOI:** 10.3390/biom14060620

**Published:** 2024-05-24

**Authors:** Li Liu, Zhiling Li, Baiyan Yang, Xiaoqing Jia, Shige Wang

**Affiliations:** 1 Outpatient Department of Anhui Medical University First Affiliated Hospital, The First Affiliated Hospital of Anhui Medical University, No. 120 Wanshui Road, Hefei High-Tech Zone, Hefei 230000, China; 2 School of Materials and Chemistry, University of Shanghai for Science and Technology, No. 516 Jungong Road, Shanghai 200093, China

**Keywords:** hydrogel, polyamidoamine dendrimer, drug delivery, tissue engineering, nanohydrogel

## Abstract

Hydrogels are three-dimensional crosslinked functional materials with water-absorbing and swelling properties. Many hydrogels can store a variety of small functional molecules to structurally and functionally mimic the natural extracellular matrix; hence, they have been extensively studied for biomedical applications. Polyamidoamine (PAMAM) dendrimers have an ethylenediamine core and a large number of peripheral amino groups, which can be used to engineer various polymer hydrogels. In this review, an update on the progress of using PAMAM dendrimers for multifunctional hydrogel design was given. The synthesis of these hydrogels, which includes click chemistry reactions, aza-Michael addition, Schiff base reactions, amidation reactions, enzymatic reactions, and radical polymerization, together with research progress in terms of their application in the fields of drug delivery, tissue engineering, drug-free tumor therapy, and other related fields, was discussed in detail. Furthermore, the biomedical applications of PAMAM-engineered nano-hydrogels, which combine the advantages of dendrimers, hydrogels, and nanoparticles, were also summarized. This review will help researchers to design and develop more functional hydrogel materials based on PAMAM dendrimers.

## 1. Introduction

A dendrimer is a synthetic, nanoscale, spherical, highly branched, monodisperse macromolecule [1,2,3]. Its molecular structure, which consists of an interior core, interior repeating units, and exterior terminal groups, has a high degree of geometric symmetry [4,5]. The molecular size, generations (Gx, x = 0.5, 1, 1.5, 2, 2.5…), and function group density can be precisely controlled, making dendrimers a hot research topic in various fields [6,7,8,9]. Typically, dendrimers can be synthesized using the divergent method, the convergent method, and the combined divergent–convergent method [10,11,12]. In recent years, with the development of chemical synthesis and characterization technology, scientists have synthesized a series of dendrimers, such as polyamidoamine (PAMAM) dendrimers, polypropyleneimine dendrimers, peptide dendrimers, glycan dendrimers, etc. [13,14,15,16,17,18,19].

As typical representatives, PAMAM dendrimers have been successfully synthesized for several decades, and dozens of PAMAM dendrimers (G1–G10) have been made commercially available [20,21]. The nanosize, shape, and structural features of PAMAM change as the number of generations increases, and the number of peripheral amino groups at the end of PAMAM increases exponentially as the number of generations increases, resulting in strong positive charges on its surface [22]. These positive charges can be complexed with DNA or RNA through electrostatic interaction and further regulate their in vivo distribution and cellular uptake, thus realizing the delivery and transfection of DNA or RNA [23,24,25]. Another typical feature of PAMAM is that its structure is hydrophobic on the inside and hydrophilic on the outside surface [26,27], allowing PAMAM to be efficiently loaded with both hydrophilic and hydrophobic drugs [28] or proteins [29]. In particular, the loading of hydrophobic drugs has been a bottleneck in clinical practice [30].

Hydrogels are three-dimensional crosslinked functional materials with water-absorbing and swelling properties [31,32]. Typically, hydrogels are extremely hydrophilic and have excellent water retention and adsorption properties. Therefore, many hydrogels can store a variety of hydrophilic functional small molecules to structurally and functionally mimic the natural extracellular matrix [33,34]. In addition, hydrogels possess an easily modifiable surface with abundant functional groups, which can be physically or chemically decorated to induce many attractive physicochemical properties, including flexibility, softness, biodegradability, and biocompatibility [35,36]. In most cases, hydrogels can be prepared via physical or chemical approaches [37,38,39,40]. The chemical preparation of hydrogels commonly involves the use of certain crosslinking agents to evoke a crosslinking reaction with polymers that have specific functional groups. Furthermore, the chemical preparation of hydrogels usually requires the involvement of an activator or initiator [41,42]. Specifically, by choosing suitable crosslinking agents, functional molecules such as drugs can be simultaneously loaded along with the hydrogel formation. This feasible form of synthesis endows hydrogels with functions like antimicrobial and antioxidant [43], self-healing [44], stimulus responsiveness [45], adhesion [46], hemostasis [47], and wound healing [48], which can further enhance the applicability of hydrogels in diverse fields.

**Figure 1:**
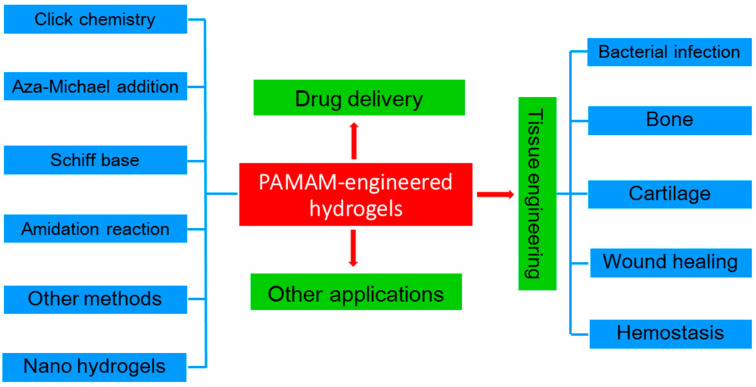
Illustration of the potential applications of biopolymer hydrogels in biomedical fields.

Here, the present work wishes to provide an update on the progress of using PAMAM dendrimers for multifunctional hydrogel design by considering recently published works (primarily from the past 10 years, from 2015 to the present). The applications of different biopolymer hydrogels in biomedical fields have been extensively reviewed and discussed before [35,49,50,51,52]. However, there are very few reviews that provide a comprehensive overview of the biomedical applications of PAMAM-dendrimer-engineered hydrogels [53]. Indeed, due to the limited availability of PAMAM, studies on PAMAM-dendrimer-engineered hydrogels are relatively limited. This review paper covers the synthesis of these hydrogels, which primarily involves click chemistry reactions, aza-Michael addition, Schiff base reactions, amidation reactions, enzymatic reactions, and radical polymerization (Figure 1). Then, the research progress in terms of their application in the fields of drug delivery, tissue engineering, drug-free tumor therapy, and other related fields is discussed in detail. In addition, PAMAM-engineered nano-hydrogels (NGs), which can combine the advantages of dendrimers, hydrogels, and nanoparticles [54], are also briefly summarized. For dendrimer-engineered NGs, since a critical review on dendrimer-based NGs for cancer nanomedicine applications was published in 2022 [55], this review only covers the most recent progress in PAMAM-engineered NGs. Finally, we describe the challenges faced by PAMAM-engineered hydrogels in practical applications. This review will help researchers to design and develop more functional hydrogel materials based on PAMAM. This review primarily aims to provide a comprehensive literature overview, highlighting the latest advancements in the fields of utilizing PAMAM dendrimers for multifunctional hydrogel design and their diverse applications in drug delivery, tissue engineering, drug-free tumor therapy, and related domains. It is important to note that this review does not delve into the clinical applications or commercial insights within the field.

## 2. PAMAM-Crosslinked Hydrogels for Drug Delivery

To date, hydrogels have been considered promising candidates for drug delivery owing to their superior physicochemical properties, good biocompatibility, and sustained drug release [56,57,58]. The drug loading of hydrogels can be feasibly accomplished by directly mixing certain drugs into the precursor solution for hydrogel formation (one-step strategy) or firstly loading them with PAMAM and then mixing them with the hydrogel precursor solution (two-step strategy; see Table 1).

### 2.1. Click Chemistry Reaction

**Figure 2:**
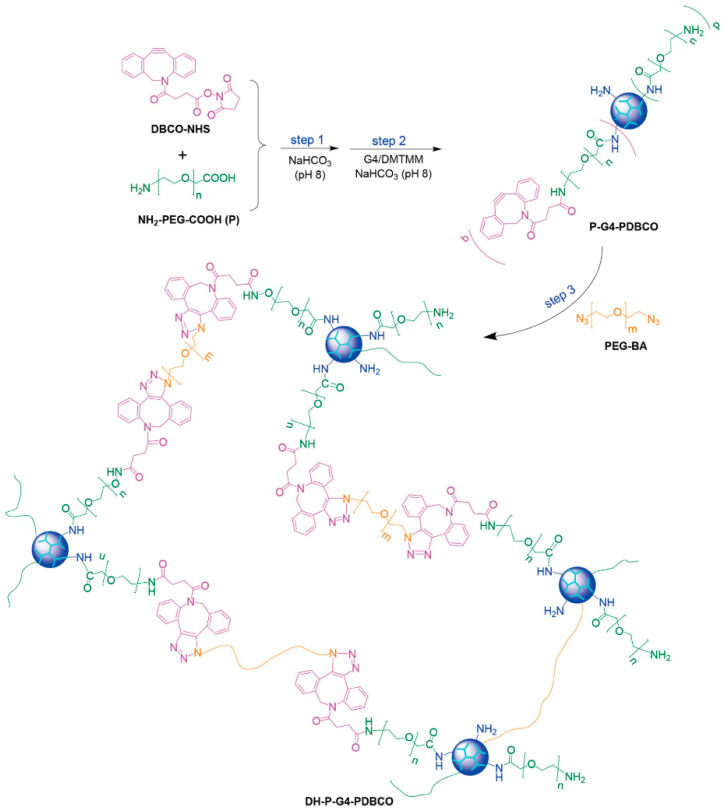
The synthetic procedures of DH-P-G4-PDBCO hydrogel (DMTMM: 4-(4,6-dimethoxy-1,3,5-triazin-2-yl)-4-methylmorpholinium chloride). Reproduced with permission from [59].

Click chemistry reactions have a high selectivity and high yield and do not require complex reaction and processing conditions. Using “click chemistry”, Yang and colleagues constructed a novel biorthogonal dendrimer hydrogel based on copper-free click chemistry (Figure 2). In this report, the authors first functionalized a PAMAM dendrimer G4 with dibenzocyclooctyne (DBCO) to introduce “clickable” dendritic macromonomers, which was then mixed with PEG bisazide (PEG-BA) [59]. In an aqueous solution, the azide and dibenzocyclooctyne underwent strain-promoted azide–alkyne cycloaddition and rapidly transformed into a dendrimer–PEG crosslinked hydrogel (DH-P-G4-PDBCO) without the addition of any catalyst. Then, 5-fluorouracil was directly dissolved in the mixed solution of PEG-BA and G4-dibenzocyclooctyne, which was directly entrapped in the hydrogel upon its formation. After intratumoral injection, the hydrogel/5-fluorouracil significantly improved the survival rate of mice with tumors by reducing tumor cell proliferation and angiogenesis and promoting tumor cell death.

As a starburst polymer, PAMAM can enhance the solubility of and control the release of hydrophobic drugs, showing advantages over conventional drug carriers. For instance, the water solubility of diflunisal, a nonsteroidal anti-inflammatory drug, can be improved 129–193-fold with the addition of a PAMAM dendrimer (15 mg/mL). Therefore, a vinyl-sulfone-functionalized PAMAM G5 dendrimer was crosslinked with thiolated PEG to form a hydrogel, which successfully encapsulated a high payload of diflunisal and controlled its release (about 87% of the diflunisal was released within 72 h) [60]. In another study, Bi et al. developed a PAMAM dendrimer and PEG-derived hydrogel to solubilize and facilitate the sustained delivery of hydrophobic silibinin, methotrexate, and camptothecin [61]. The vinyl-sulfone-functionalized PAMAM (45 mg/mL) increased the solubility of silibinin, methotrexate, and camptothecin 37-fold, 10-fold, and 4-fold, respectively. Then, the functionalized PAMAM dendrimer and thiolated PEG were crosslinked into a hydrogel, which successfully controlled the drug release. In water, the 4-day cumulative amount released of these hydrophobic drugs reached 80%. Viability assays of MCF7 and J82 cells revealed that all three drugs were cytotoxic to both cell lines after 2 days. Notably, the less soluble camptothecin showed higher cytotoxicity than silibinin and methotrexate in both cell lines, reaching up to 95% cell death. This research provides further evidence that PAMAM-engineered hydrogels may act as promising platforms for sustained hydrophobic drug delivery and potential tumor therapy.

### 2.2. Aza-Michael Addition

**Figure 3:**
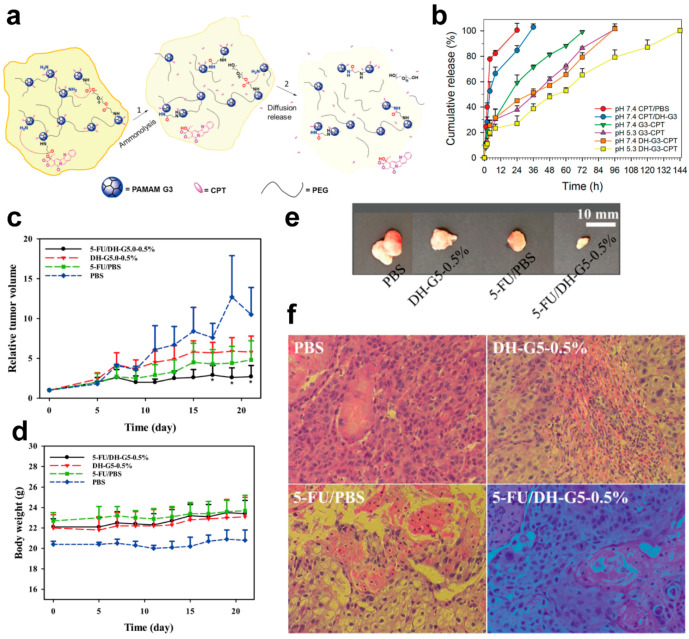
(**a**) The ammonolysis-induced cleaving and the CPT-releasing mechanism of DH-G3-CPT. (**b**) In vitro drug release results at body temperature (*n* = 3). Panels a and b were reproduced with permission from [63]. (**c**) Relative tumor volume of 5-FU/DH-G5-0.5%-treated mice (* *p* < 0.05). (**d**) Body weight change in 5-FU/DH-G5-0.5%-treated mice. (**e**) Extracted tumor images of 5-FU/DH-G5-0.5%-treated mice. (**f**) H&E staining of tumor tissues after different treatments (magnification, 200×). Panels (**c**–**f**) were reproduced with permission from [65].

Aza-Michael addition is a highly efficient reaction that can form C–N bonds [71]. CPT, as a hydrophobic anticancer drug that can inhibit topoisomerase I activity [72], was modified to include acrylate end groups and grafted onto the surface of a PAMAM dendrimer G3 via aza-Michael addition. The resultant G3/CPT was further conjugated with PEG-DA to construct a crosslinked self-cleavable dendrimer hydrogel [63]. The cleaving of CPT can be attributed to the ammonolysis of the ester; therefore, the self-cleaving is pH-tunable (Figure 3a). In particular, since the CPT loading was based on a chemical conjugation, the CPT release was prolonged (Figure 3b). The tumor inhibition effects of 5-FU/PBS are not significant. In contrast, 5-FU/DH-G5-0.5% significantly inhibited tumor growth (Figure 3c). During the treatment, the mice showed no obvious body weight loss (Figure 3d). The tumor images (Figure 3e) further indicate the therapeutic efficiency against tumors. H&E staining (Figure 3f) suggests that 5-FU/DH-G5-0.5% resulted in a high level of massive tumor cell remission. Therefore, the injectable hydrogel may act as a sustained drug delivery platform for localized tumor chemotherapy.

In another study, via aza-Michael addition of a PAMAM dendrimer G5 and PEG-DA, the same research group developed a mildly crosslinked hydrogel. Using ^1^H nuclear magnetic resonance spectroscopy, it was found that the mildly crosslinked dendrimer hydrogel was able to unionize brimonidine tartrate to form and encapsulate a brimonidine-free base, which afforded enhanced corneal permeation and sustained release of the brimonidine tartrate [64]. Dendrimer hydrogels formed from the aza-Michael addition reaction can also be used to physically load chemotherapeutics. For example, the nucleophilic surface amines of a PAMAM dendrimer G5 were reacted with an α, β-unsaturated ester of PEG-DA via a catalyst-free aza-Michael addition reaction [65]. To physically load the chemotherapeutics, the anticancer drug 5-fluorouracil was directly mixed with the precursor solution and entrapped in the hydrogel upon its formation. Following intratumoral injection, this new class of injectable dendrimer hydrogel efficiently inhibited tumor growth. By combining aza-Michael addition with the water-in-oil (w/o) inverse microemulsion method, they further explored PAMAM-G5-cross-linked PEG-DA microgels [66]. Using this protocol, CPT can be encapsulated into the aqueous dendrimer solution and transformed into a CPT-loaded dendrimer microgel. The microgels are safe in vitro and in vivo and can smoothly enter the cells as particles and release the CPT following zero-order release kinetics.

### 2.3. Schiff Base Reactions

**Figure 4:**
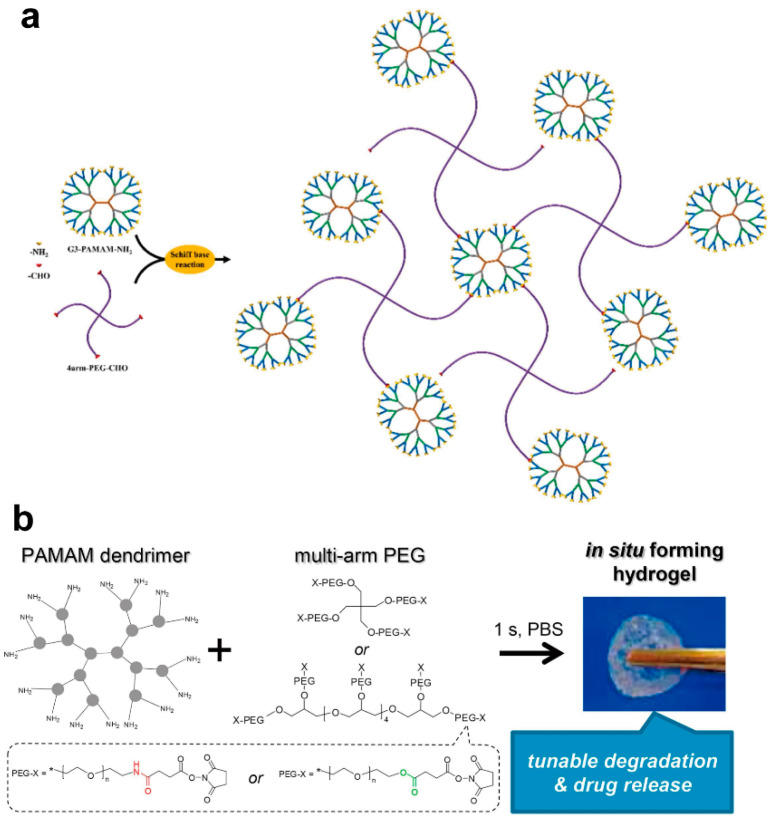
(**a**) Scheme of hydrogel formation via Schiff base reaction between NH_2_ groups on PAMAM dendrimers and CHO groups on four-arm PEG. Reproduced with permission from [73]. (**b**) The scheme of in situ formation of chemically crosslinked PEG/PAMAM hydrogels. Reproduced with permission from [68].

Typically, the NH_2_ groups on PAMAM can react with the CHO groups of certain polymer chains via a Schiff base reaction. Li et al. reported the construction of PAMAM (G1, G3, and G5) and aldehyde-terminated four-arm PEG hydrogels (Figure 4a) [73]. Their results suggest that the generation of PAMAM can substantially influence the mechanical properties of PAMAM-Tetra-PEG hydrogels, and G3 and G5 can produce high-tough hydrogels with fatigue resistance (~300 cycles of loading–unloading under strain of 90%) and a high compression strength (0.872 MPa). A typical application of PAMAM-crosslinked hydrogels is for dermal drug delivery [74]. Many hydrogels are injectable and can fit well on the surface of the skin or even into skin tissues for irregularly shaped wounds. For example, a pH-responsive hydrogel film was formed from a three-dimensional reticulated dendrimer megamer and glutaraldehyde, with a polytetrafluoroethylene flat sheet as the substrate. The glutaraldehyde was used to crosslink the PAMAM to prepare the dendrimer megamer and was further conjugated with the dendrimer megamer to prepare the hydrogel film. The unique megamer architecture and chemical constitution enabled the hydrogel film to load both hydrophilic and lipophilic substances. A rabbit skin irritation test illustrated that the hydrogel did not cause any toxicity and allowed for controlled ketoprofen release [67].

### 2.4. Amidation Reaction

Upon reaction with the N-succinimidyl ester of multi-armed PEG (four-arm or eight-arm), PAMAM G2 quickly forms chemically crosslinked PEG/PAMAM hydrogels in situ through an amidation reaction. The linkers between the PEG arms and their terminal N-succinimidyl ester groups can be amides or esters [68]. The PAMAM and PEG solutions transform into hydrogels within seconds of mixing them. The storage moduli of PEG/PAMAM hydrogels increased with their crosslinking density and reached 2.3 kPa when four-armed PEG was used (Figure 4b). Amide-linked hydrogels with ester linkages showed a faster degradation rate than amide-linked hydrogels (2 days versus several months). The release of fluorescein isothiocyanate-dextran (4 × 10^3^ or 2 × 10^6^ g/mol) from amide-linked hydrogels featured an initial burst and then diffusion-controlled release. In contrast, the release of fluorescein isothiocyanate-dextran (dextran: 2 × 10^6^ g/mol) from ester-containing hydrogels was tunable, depending on the ratio between the amide linkages and the ester. With tunable degradation, mechanical, and release properties, these non-toxic PEG/PAMAM hydrogels formed in situ hold promise as a platform for sustained drug delivery.

### 2.5. Other Reaction Methods

Except for antitumor drugs, other kinds of antiinflammation drugs or anticoagulants can also be loaded with PAMAM dendrimers. For instance, via an enzymatic reaction, tyramine-conjugated tetronic and p-hydroxyphenyl-acetic-acid-functionalized PAMAM G3 were mixed to form a cationic hydrogel in the presence of the horseradish peroxidase enzyme and hydrogen peroxide and the anticoagulant heparin loaded [69]. Cationic hydrogels can sustainably release the anionic anticoagulant heparin, which finds diverse application opportunities for coating stents or blood-contacting devices. Nyström et al. disclosed the relationship between the amount of PAMAM and the structure, swelling properties, and drug release characteristics of hydrogels [70]. In this study, N-isopropy-lacrylamide, N,N′-methylenebis(acrylamide), and PAMAM G6 were dissolved in water and mixed with ammonium persulfate and N,N,N′,N′-tetramethylethylene-diamine. After reaction at 20 °C for 16 h, a thermo-responsive poly(N-isopropylacrylamide) hydrogel was formed. This study elucidates that the PAMAM entities increase the swelling and loading capacity and expand the poly(N-isopropylacrylamide) hydrogel into a more homogeneous state. Via varying the amounts of the PAMAM, improved drug delivery is achieved. In another scheme, Pistone and co-workers prepared a chitosan–dendrimer–hydroxyapatite hydrogel by covalently grafting chitosan powder with a hyperbranched PAMAM dendrimer, followed by in situ precipitation of the hydroxyapatite and gelification. They demonstrated the ability to adjust the mechanical properties of hydrogels to achieve the desired drug release kinetics. This study emphasizes the importance of tailored mechanical properties in optimizing drug delivery systems for enhanced therapeutic efficacy, providing insights into the design and optimization of hydrogel formulations with tunable rheological properties [56].

## 3. PAMAM-Crosslinked Hydrogels for Tissue Engineering

### 3.1. Bacterial Infection Treatment

**Figure 5:**
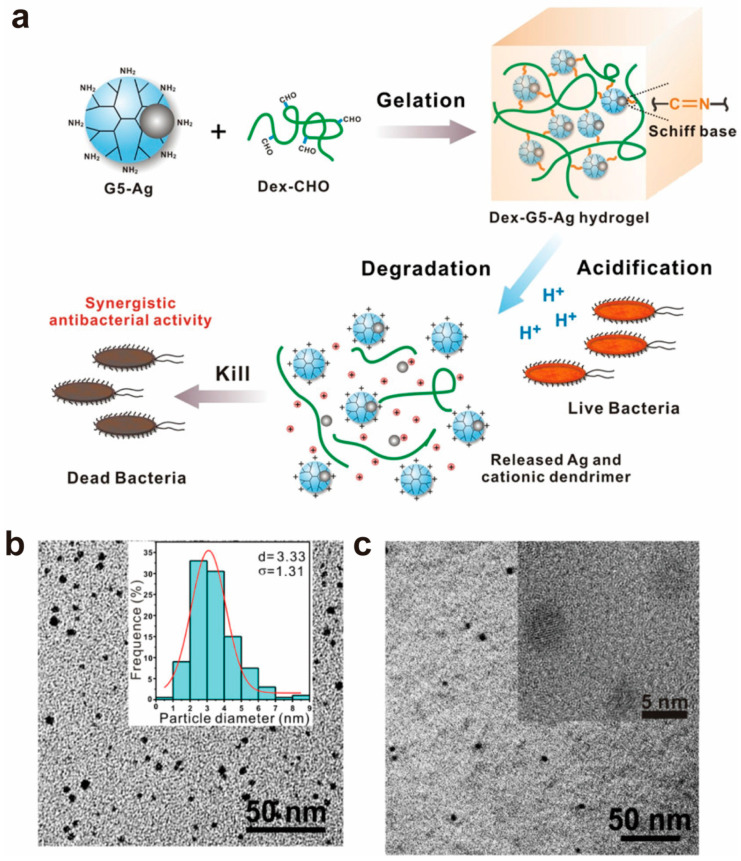
(**a**) The design strategy for Dex-G5-Ag hydrogel. The hydrogel is pH-responsive and degradable in acidic circumstances to release Ag and G5 for synergistic antibacterial applications. (**b**) Transmission electron microscope image and size distribution of G5-Ag (30) NPs. (**c**) Transmission electron microscope image of the Dex-G5-Ag (30) gel. Reproduced with permission from [81].

Local bacterial infection is a serious threat to human health and remains challenging in clinics [75,76]. Conventional antibiotics have short-term antibacterial activity and may cause bacterial resistance [77,78]. Compared with various existing antibiotics and antimicrobial drugs, PAMAM dendrimers are novel antimicrobial molecules, and their antimicrobial effect may be related to how many terminal amino groups they have [79]. It has been found that high-generation PAMAM (e.g., G3 and G5) possesses significant antimicrobial activity in vitro and can significantly inhibit the growth of *Pseudomonas aeruginosa* and *Staphylococcus aureus* [80]. Cheng et al. studied the broad-spectrum antibacterial activity of a PAMAM-dendrimer-engineered composite hydrogel. They designed a pH-responsive composite hydrogel via a Schiff base linkage between PAMAM and oxidized polysaccharides (e.g., dextran) [81]. Before the hydrogel’s formation, PAMAM G5 was first encapsulated with silver nanoparticles (Figure 5a,c). In this hydrogel, both the G5 and silver species acted as antibacterial components (Figure 5d). In particular, the acidity generated by growing bacteria could trigger the release of the G5 and silver species, which exhibited a synergistic effect in terms of their antibacterial activity against both Gram-positive (*Staphylococcus epidermidis* and *Staphylococcus aureus*) and Gram-negative (*Escherichia coli* and *Pseudomonas aeruginosa*) bacteria. Compared with a commercial silver hydrogel, the biocompatible hydrogel exhibited better in vivo antibacterial efficacy against *Staphylococcus aureus* infection at an identical silver concentration, providing a potential method for designing intelligent hydrogels for local bacterial infection treatment.

As a kind of natural cationic polysaccharide, chitosan has good biosafety and biodegradability. Due to the presence of protonated amino groups in its molecular chain, chitosan exhibits certain antibacterial and antioxidant properties [82,83,84]. In addition, the reactive amino groups in chitosan’s units can participate in grafting, crosslinking, and polymerization reactions; therefore, chitosan is often used in the preparation of multifunctional biomaterials [85,86]. Using glutaraldehyde as a crosslinking agent, He et al. prepared PAMAM-dendrimer-modified quaternary chitosan hydrogels [82,83]. In these studies, the PAMAM G2 hydrogels were prepared via a divergent method using ethylenediamine as the core. The hydrogels featured a highly porous three-dimensional network structure. Swelling tests of the hydrogels showed good swelling and pH sensitivity. An increase in the PAMAM content or quaternization resulted in improved swelling properties. Furthermore, it was found that hydrogels with lower concentrations of the crosslinker or lower concentrations of quaternary ammonium chitosan had better swelling properties. Further, the antimicrobial results showed that the hydrogels exhibited better antimicrobial activity against both *Staphylococcus aureus* and *Escherichia coli* as the PAMAM content, the quaternary chitosan concentration, or the crosslinking agent concentration increased. Therefore, composite hydrogels with a good swelling ability and good antibacterial activity have potential applications in the field of antibacterial materials.

### 3.2. Bone Tissue Engineering

**Figure 6:**
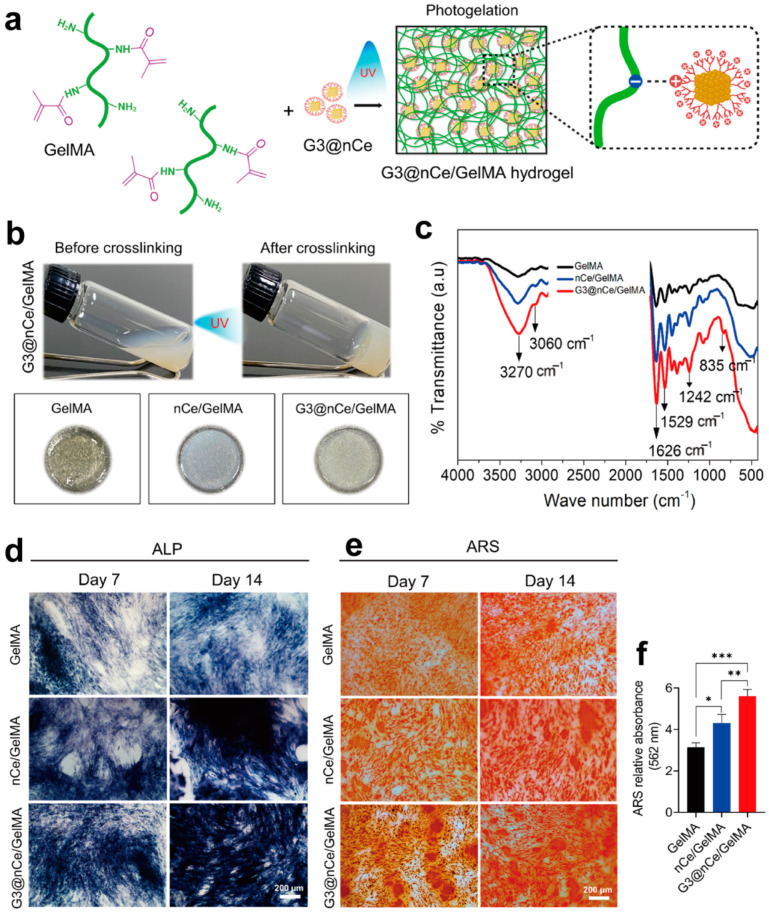
(**a**) Schematic illustration of the photo-encapsulation of G3@nCe into GelMA hydrogel and interactions between G3@nCe and GelMA. (**b**) G3@nCe/GelMA before and after crosslinking and different hydrogels after photo-gelation. (**c**) FTIR spectra of different hydrogels. (**d**) Alkaline phosphatase (ALP) activity of mesenchymal stem cells on days 7 and 14 of differentiation. A darker color suggests a higher ALP activity and differentiation degree. (**e**) Mineralization of mesenchymal stem cells observed using Alizarin Red staining (ARS). A darker color suggests a higher ALP activity and differentiation degree. (**f**) Colorimetric estimation of mineralization on day 14 (n = 3, *** *p* < 0.001, ** *p* < 0.01, * *p* < 0.05). Reproduced with permission from [89].

Bone defects are challenging clinical problems that entail the microenvironment boosting stem cell functions and alleviating oxidative-stress-induced inflammation. Hybrid and multifunctional hydrogels hold great promise in promoting the repair of bone defects [87,88]. In a study, nanoceria (nCe) was hydrothermally prepared and surface-functionalized independently with citric acid according to a carboxylation reaction. After that, the nCe was conjugated with G3 to form G3@nCe (Figure 6a). To prepare the G3@nCe-engineered hydrogel, gelatin methacryloyl was mixed with the photoinitiator 2-hydroxy-1-[4-(2-hydroxyethoxy)-phenyl]-2-methyl-1-propanone and nCe. The hydrogel’s formation was triggered by UV light irradiation (Figure 6b) and characterized using Fourier transform infrared spectroscopy (FTIR, Figure 6c), etc. [89]. The study’s conclusions proved that the G3@nCe could enhance the gel’s mechanical properties and enzymatic ability to clear reactive oxygen species and survive in spite of hydrogen-peroxide-induced high oxidative stress. Moreover, the hydrogels increased the growth and osteogenic differentiation of bone marrow mesenchymal stem cells (Figure 6d–f). They demonstrated an effective bone regeneration capacity in a rat critical-sized bone defect model when subcutaneously implanted.

Biocompatible and mechanical tunable hydrogels can influence the behavior of and influence the differentiation of bone marrow mesenchymal stem cells [90,91]. The mechanical properties of PEG-modified polyamidoamine dendrimer hydrogels and their effects on mesenchymal stem cell differentiation were investigated. To regulate the differentiation of stem cells, Bi et al. developed a multifunctional hydrogel by crosslinking vinyl-sulfone-functionalized, multi-armed, thiolated PEG with a PAMAM dendrimer through thiol–ene Michael addition in aqueous conditions [92]. By changing the PEG and PAMAM dendrimer concentrations, the gelation time and stiffness of the hydrogel were tunable; therefore, a hydrogel with a well-defined structure and adjustable mechanical properties was created. The study found that the mesenchymal stem cells were alive in the as-designed hydrogel after 2 days of incubation. Importantly, soft hydrogel (with a complex modulus of 77 Pa) tends to induce more adipogenic differentiation, while more osteogenic differentiation is observed in hard hydrogel (with a complex modulus of 5663 Pa). This phenomenon may potentially be important in the construction of therapeutic hydrogels for bone tissue engineering applications. This study highlights the importance of mechanical cues in directing cell behavior and tissue regeneration processes.

Artificial extracellular matrices of mammalian tissues are needed clinically; however, their production remains a challenge. The current poly(lactide)-b-poly(ethylene glycol)-b-poly(lactide)-derived hydrogels have adjustable degradation properties and good biocompatibility. However, the limitations of this polymer include poor mechanical properties due to its excessive volume expansion after crosslinking and its lack of bioactivity. To solve the above problems, hydrogels made of a linear copolymer of poly(lactic acid)-b-poly(ethylene glycol)-b-poly(lactic acid) with an acrylate end group (PEG-LA-DA), a linear copolymer of poly(ethylene glycol) with an arginine-glycine-(aspartic acid)-(D-tyrosine)-cysteine end group (AGPR), and a PAMAM dendrimer G4 peripherally modified with acryloyl were prepared [93]. Various crosslinking sites on G4 can increase its crosslinking density at lower concentrations. Spherical dendrimers can limit swelling and improve mechanical properties. Moreover, multiple end groups on the G4 introduce functional groups into the system at the nanoscale level. The experimental results show that this novel hydrogel system can mimic the extracellular matrix of native tissues. The introduction of AGPR into the hydrogel promoted the formation of a highly porous network and effectively improved its mechanical stiffness and reduced its swelling behavior. This kind of PAMAM-engineered hydrogel supports bone marrow mesenchymal stem cell proliferation and differentiation without cytotoxic effects and thus can be used as a model material for tissue engineering applications.

In another study, a high-performance hydrogel was designed and produced by integrating two nanospherical assemblies consisting of a combination of PAMAM G4 modified with a bioactive fragment and 8-arm PEG terminally modified with 3,4-dihydroxy-L-phenylalanine (DOPA) (OPD). The OPD was prepared according to the hydrochlorination of 4-nitrophenyl-(3-(pyridin-2-dithioalkyl)propyl)-carbonate-activated dopamine with thiol-terminated eight-armed PEG. Methoxy-PEG-succinimidyl carbonate (mPEG1000-NHS) was linked onto the surface of the PAMAM G4 and maleimide-PEG-succinimidyl carbonate (MAL-PEG5000-NHS) and covalently conjugated with the arginine-glycine-(aspartate)-(D-tyrosine)-cysteine bioactive peptide through an effective Michael addition reaction to enhance the biocompatibility and solubility. It was demonstrated that the hydrogels had a three-dimensional pore-like structure, a higher mechanical strength, and a low and stable swelling rate. Moreover, the hydrogel obtained with an OPD concentration of 15% and a ratio of 1/2 with the biomodified G4 had the best performance. Its in vivo bone regeneration application potential was validated in a mouse calvarial critical-sized defect model, which confirms that this hydrogel could be used as a substrate for a potential clinical tissue engineering scaffold [94].

### 3.3. Cartilage Tissue Engineering

Cartilage defects have led to an urgent need to design regenerative medicine for cartilage repair. Designing biocompatible and injectable cell delivery vehicles that can efficiently induce the chondral differentiation of stem cells is an essential method for cartilage regeneration [95,96]. In this field, a photo-crosslinked gelatin methacryloyl hydrogel containing PAMAM-MA as a filler to strengthen its plasticity was designed as an adipose-derived stromal/stem cell delivery scaffold for cartilage repair [97]. These adipose-derived stromal/stem cells were directly resuspended in the hydrogel prepolymer solution and encapsulated in the hydrogel for its formation. Both the in vitro adipose-derived stromal/stem cell three-dimensional culture and in vivo articular cartilage defect repair studies confirmed that the hybrid hydrogel had a sufficient ability to promote cartilage regeneration, providing new insights into the design of stem cell delivery scaffolds.

### 3.4. Wound Healing

Wound healing is a complex biological event, with overlapping stages to obtain complete re-epithelization [98,99]. The current wound treatment methods are diverse and typically include physical, chemical, or biochemical ones. Dendrimer-based hydrogels have found application for full-thickness wound management. For example, hesperidin can be used for the treatment of different kinds of wounds. However, its poor water solubility leads to lower bioavailability and has limited its therapeutic applications. In a recent study, Kesharwani et al. incorporated hesperidin into the inner core of PAMAM to increase its water solubility and bioavailability. Then, sodium alginate solution was mixed with a chitosan and hesperidin–PAMAM solution to obtain hydrogel bandages [85]. With proven biocompatibility, evaluation of its therapeutic efficacy showed that the hydrogel promoted the generation of degenerated neutrophils and eosinophils, suggesting better wound contraction activity than the control group in a full-thickness wound animal model. By varying the hesperidin–PAMAM content, it was reported that 10% hesperidin in the formulation was most suitable for treating skin-related injuries or wounds.

### 3.5. Hemostasis

**Figure 7:**
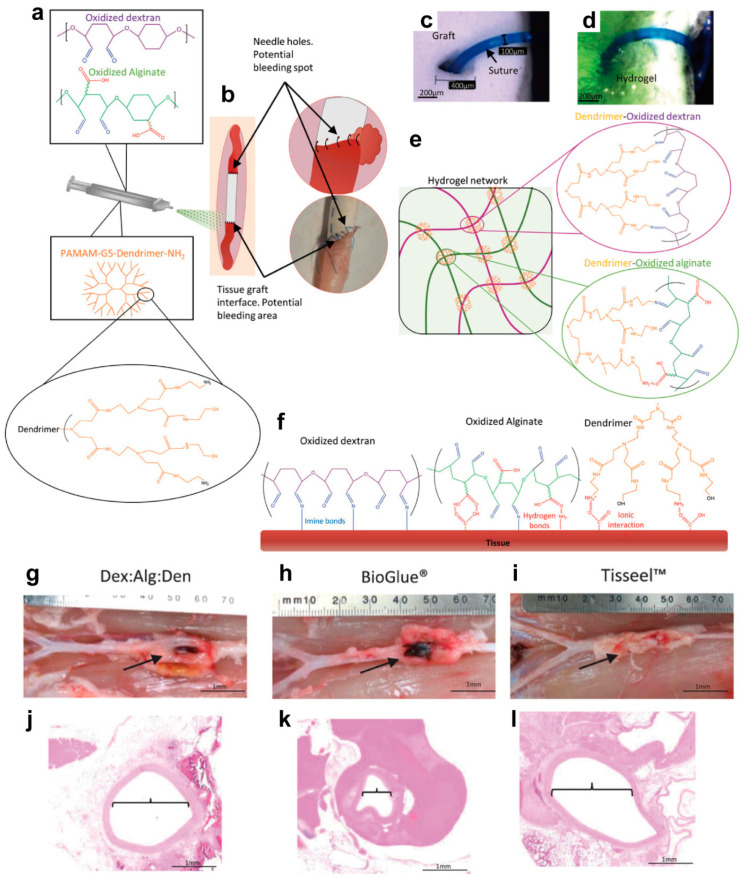
(**a**) Illustration of the dual-barrel syringe (chamber 1: oxidized dextran and oxidized alginate; chamber 2: PAMAM dendrimer G5). (**b**) Illustration of the interface between the vessel and the vascular graft. (**c**) Micrography of the ePTFE graft with a single suture point. (**d**) The same suture is depicted following spraying to prevent leakage. (**e**) Illustration of the hydrogel crosslinking structure. (**f**) Illustration of the tissue adhesions. (**g**–**i**) Aorta puncture site for 15%Dex:5%Alg:30%Den (**g**), BioGlue (**h**), and Tisseel (**i**). (**j**–**l**) H&E imaging shows the vessel diameter for 30%Dex:5%Alg:15%Den (**j**), BioGlue (**k**), and Tisseel (**l**); scale bar = 1 mm. Reproduced with permission from [102].

Complications related to bleeding after vascular surgery are one of the most common complications in vascular surgery, which seriously threaten the health of patients [100]. Commercially available hemostatic materials mainly rely on forming covalent bonds between the hemostatic materials and the tissue surface to achieve adhesion [101]. Hydrogels have essential applications as potential hemostatic materials. Yet none of the current commercial hemostatic materials have desirable properties for affording an adequate vascular seal. In a former study, researchers developed a spray-type hydrogel for the immediate closure of vascular anastomoses to improve the safety and efficiency of vascular surgery (Figure 7a,b). The hydrogel formation was based on a Schiff base between oxidized alginate and oxidized dextran with PAMAM dendrimers (Figure 7e), which rapidly and firmly adhere to tissue surfaces (Figure 7c,d) utilizing electrostatic interactions and covalent bonding (i.e., Schiff base linkages, Figure 7f). The key results disclosed that this hydrogel demonstrated excellent containment properties and biocompatibility in ex vivo experiments. It can withstand high pressures beyond physiological levels (~300 mmHg), effectively prevents bleeding in animal models, and outperforms similar products available on the market. In addition, biocompatibility testing in vivo and ex vivo demonstrated the potential value of the hydrogel for reducing complications in vascular surgery, especially at difficult-to-seal tissue–graft interfaces. In a rabbit model, on day 5, the oxidized dextran (Dex): oxidized alginate (Alg): dendrimer (Den) sealant (Figure 7g) and BioGlue (Figure 7h) presented a dark gold color, which was related to the crosslinked imine bonds. Fibrin glue showed a white and fibrous appearance, which was related to the enzymatic crosslinking (Figure 7i). In the histological analysis (Figure 7j–l), while BioGlue showed severe aorta narrowing, both the sealant and the fibrin glue displayed an open lumen [102].

As a qualified hemostatic dressing, a hydrogel should have strong tissue adhesions and mechanical properties to withstand blood pressure. The tissue adhesion can originate from hydrogen bonding or various types of chemical bonding between the tissue and the hydrogel [103,104]. Zhao et al. designed in situ injectable hydrogels containing oxidized dextran and thiol-functionalized PAMAM dendrimers under simulated physiological conditions, presenting an innovative approach to enhancing the mechanical strength of hydrogels through double-crosslinking with PAMAM and oxidized dextran [105]. The resulting hydrogels exhibited high mechanical strength and injectability, making them promising candidates for various biomedical applications requiring robust scaffolds or delivery systems. Under the optimized conditions (e.g., a moderate PAMAM concentration of 10%), the adhesive strength of the hydrogels were 2.4 times higher than that of commercially available fibrin glue. The hydrogels featured a double-crosslinked structure involving the Schiff base bonds and disulfide bonds. It was found that the generation of PAMAM, the concentration of PAMAM, and the amino group density on the surface are the main factors affecting hydrogel formation, and a high amino group density on the surface may enable fast gelation of solution and hydrogel formation. However, too high a density of amino groups on the surface can result in a hydrogel with increased brittleness. Moreover, the double-crosslinked structure, the density of the disulfide bonds, and the hydrogen bonds in these hydrogels may also change their storage moduli. Equally, the gelation process does not require any cytotoxic crosslinkers, making these hydrogels nontoxic. Therefore, the L929 fibroblast cells could attach easily to the hydrogel surface and proliferate well. These injectable and double-crosslinked PAMAM-engineered hydrogels showcase the possibilities of adjusting the adhesive strength of hydrogel hemostatic dressings.

The mechanical properties of hydrogels are usually related to their crosslinking structure. Graphene oxide (GO) is a 2D nanomaterial with a high specific surface area, a high fracture strength, and a high modulus. GO produced using Hummers’ method has a high density of surface carboxyl groups and therefore can be incorporated with PAMAM dendrimers. Chen et al. reported creating a PAMAM-GO nanocomposite hydrogel by mixing and stirring GO (produced using Hummers’ method) with PAMAM solutions. A PAMAM dendrimer G4 was synthesized using the divergent method reported by Tomalia et al. [106]. Hydrogels were mainly formed according to the electrostatic interaction between the oppositely charged PAMAM and GO. The effects of the GO and PAMAM concentrations, the transverse size of the GO nanosheets, and the degree of oxidation on the mechanical properties of the hydrogels were further explored. The results showed that the highest storage modulus of the PAMAM-GO hydrogels reached 284 kPa, which is higher than that of GO–gelatin nanocomposite hydrogels. Finally, the relationship between the crosslinking density and the storage modulus of the GO-PAMAM nanocomposite hydrogels was summarized, indicating that hydrogels with a higher modulus also have a higher crosslinking density [107].

## 4. PAMAM-Crosslinked Hydrogels for Other Applications

### 4.1. Tumor Photothermal Therapy (PTT)

Except for their use as crosslinkers, PAMAM dendrimers can also be feasibly doped into hydrogel matrices for different biomedical applications [108]. Some PAMAM dendrimer-based drug-free hydrogels are applicable to tumor therapy even without the introduction of chemotherapeutics. Cheng et al. reported the immobilization of dendrimer-encapsulated platinum nanoparticles (DEPts) with an alginate–calcium (Ca^2+^) hydrogel via electrostatic interactions [109]. This kind of PAMAM/DEPt hydrogel can be locally encapsulated in the tumor location to afford long-term and repeated PTT. More importantly, the PAMAM/DEPt hydrogel was degradable in the presence of chelates (e.g., ethylene diamine tetraacetic acid, sodium citrate, and diethylene triamine pentacetic acid). The PAMAM/DEPt hydrogel successfully prolonged the retention of the DEPts in the tumor sites and enabled repeated tumor PTT with reduced toxicity. In another study, they represented another injectable and degradable PAMAM/DEPt hydrogel, which was composed of aldehyde-modified dextran and DEPts, for the same tumor therapy purpose [110]. The hydrogel formation was based on the formation of imine bonds between the PAMAM and aldehyde-modified dextran. The photothermal effect was derived from the DEPts. Repeated PTT led to efficient tumor inhibition. After the treatment, the hydrogel was degraded due to the imine bonds’ decomposition.

Realizing the cancer-abrogating potential of miRNA is challenged by the need for efficient delivery vehicles. Artzi et al. reported a self-assembled hydrogel scaffold, produced with a dual-color RNA triple-helix structure (comprising an antagomiRNA (an oncomiRNA inhibitor) and a miR mimic (tumor suppressor miRNA)), a PAMAM dendrimer, and dextran [111]. Because the dextran aldehyde can chemically interact with and adhere to the tumor tissue amines, the hydrogel scaffold is bio-adhesive. Therefore, after two weeks, the implanted hydrogel led to nearly 90% levels of tumor shrinkage in a triple-negative tumor-bearing mouse. This study suggests that drug-free hydrogels can also be used as a qualified anticancer platform.

### 4.2. Biofabrication

To date, hydrogels have attracted extensive attention for biofabrication due to their structural similarity to the natural extracellular matrix, potential for cell encapsulation into 3D networks, and ability to provide biological-related physical and chemical cues and offer various shapes and biomechanical characteristics [112,113]. To combine the merits of natural and synthetic polymer hydrogels, Bi and co-workers designed a binary PAMAM–hyaluronan hydrogel with fast crosslinking. To facilitate hydrogel formation, vinyl sulfone groups were chemically modified at the dendrimer periphery to crosslink with the thiolated hyaluronan (thiol–ene reaction). Then, an arginylglycylaspartic acid was chemically attached to the PAMAM dendrimer to enhance the cell adhesion and promote cell proliferation [114,115]. With excellent biocompatibility, this PAMAM-engineered hyaluronan hydrogel could be a potential platform for different biofabrication applications.

### 4.3. Smart PAMAM-Engineered Hydrogels

Smart hydrogels are capable of changing their volume and physical–chemical properties in response to outside stimuli such as pH, temperature, and certain chemicals and therefore have found tremendous application potential [116,117]. In a recent study, Wang et al. designed a temperature- and pH-responsive PEG-grafted PAMAM dendrimer hydrogel. In their study, the PEG glycidyl ether (PEGOx, x = 600, 1000, and 2000) was first synthesized via the reaction of PEGx (x = 600, 1000, and 2000) and epichlorohydrin in the presence of NaOH. Then, the synthesized PAMAM dendrimer G1 [118] was reacted with PEGO–x according to constant stirring for 24 h at 45 °C. They found that the hydrogel exhibited obvious temperature sensitivity (with the critical-phase transition temperature ranging from 40 to 50 °C). Furthermore, the hydrogel exhibited obvious pH sensitivity in the pH range of 8–11. The amino groups were easily protonated in an acidic medium, resulting in a higher swelling degree, whereas in an alkaline medium, the protonated amines could be converted into neutral ones, and the swelling degree was low. Moreover, the swelling behavior of the hydrogel showed good swelling–deswelling reversibility [119].

In another study, Vivek and co-workers introduced a novel anthracene-modified PAMAM–N-isopropyl acrylamide hydrogel. There was covalent bond formation between the dendrimer’s peripheral groups and the acrylic parts of both polymers. In addition, there was a hydrogen bonding interaction between the crosslinker and the polymers. These covalent bonds and the hydrogen bonding enabled hydrogel formation [120]. The hydrogel showed excellent near-infrared-responsive self-healing properties since it contained photo-responsive anthracene units and thermo-responsive N-isopropyl acrylamide units. Further, as a filler, the silica nanosphere gave the hydrogel more mechanical strength. The hydrogel was used for super adsorption towards methyl orange from water, with an adsorption of approximately 96%. However, no cationic or neutral dyes were adsorbed. This was due to the protonation of all the amine groups in the dendrimer, and as a result, there was an electrostatic interaction with the methyl orange dye. These results taken together suggest that a PAMAM–silica/N-isopropyl acrylamide hydrogel could be a promising candidate for developing multifunctional smart materials that could be used for bioseparation.

## 5. PAMAM-Engineered Nano-Hydrogels

NGs are three-dimensional nano-networked porous structures formed of physically or chemically crosslinked amphiphilic or hydrophilic polymers, which combine the advantages of dendrimers, hydrogels, and nanoparticles [121,122]. Micro/nano-hydrogels have many unique characteristics, such as ease of administration, a large specific surface area, and drug-targeting capacities, making them competitive for different biomedical applications [123]. NGs with hydrophilic or amphiphilic surfaces can also carry different imaging agents or provide abundant functional groups for modification. NGs show good potential for improving the solubility, biocompatibility, stability, targeting, and therapeutic effects of hydrophobic drugs [124,125]. Hence, NGs have found different applications in the fields of biomedicine, drug delivery, and soft matter science.

Oxidation reactions can also be used to prepare PAMAM-based hydrogels. In a previous study, Jia et al. designed a PAMAM G4 hydrogel based on the NaIO_4_-initiated chemical crosslinking of the peripheral functional groups of the PAMAM G4 [126]. These functional groups were introduced by modifying the enzyme-responsive Ac-arg-ala-ala-asp-D-tyr-cys-NH_2_ and bioadhesive Fmoc-arg-gly-asp-cys-SH on the periphery of the PAMAM G4. Hydrogel formation occurred due to the NaIO_4_-induced reaction of the phenol groups of tyrosine contained in Ac-arg-ala-ala-asp-D-tyr-cys-NH_2_. Drug (doxorubicin) loading was achieved using an equilibrium dialysis method. In the presence of elastase, the NGs with enzyme-responsive ligands significantly decomposed to release doxorubicin in a sustained manner in vitro, demonstrating promising potential for drug delivery in cancer therapy.

**Figure 8:**
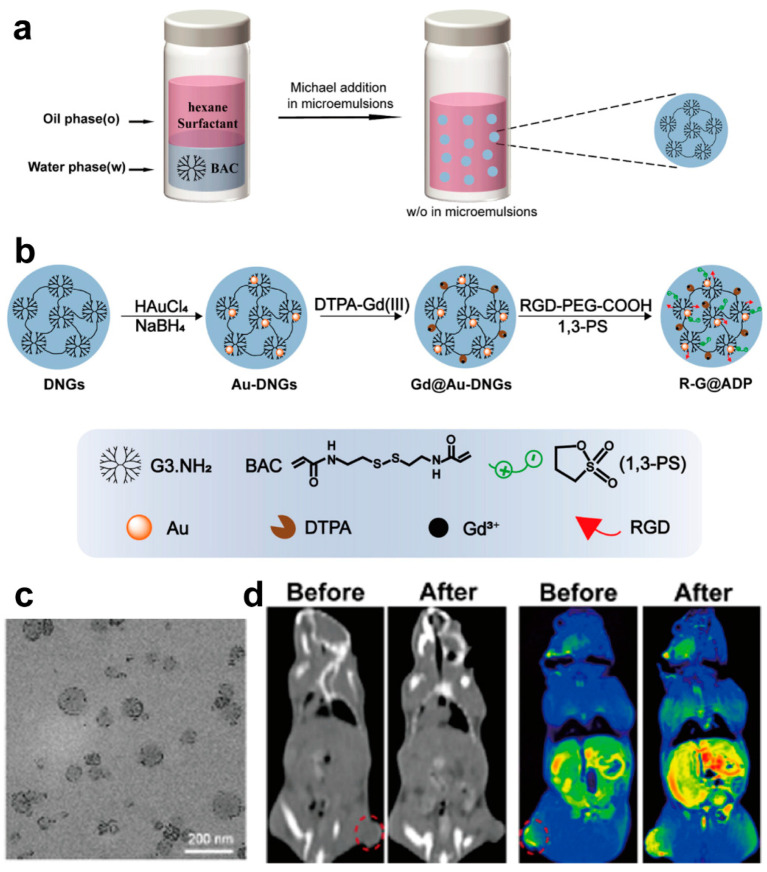
(**a**,**b**) Schematic of procedures of the (**a**) DNG and (**b**) R-G@ADP synthesis. (**c**) TEM image of the R-G@ADP. (**d**) Representative (**left**) computed tomography images and (**right**) T1-weighted magnetic resonance images of Panc-2 tumors before and 90 min after intravenous R-G@ADP injection ([Gd] = 0.01 Mol/L, [Au] = 0.1 Mol/L). Red circle: tumor area. Reproduced with permission from [127].

In the most recent research, using the inverse microemulsion method, Shen et al. prepared multifunctional PAMAM G3 NGs crosslinked with N,N′-bis(acryloyl) cystamine (DNGs) through a Michael addition reaction. Then, the DNGs were further loaded with Au NPs to produce Au-DNGs, which were liked with a diethylenetriamine penta-acetic acid (DTPA)–gadolinium (Gd) complex, PEG-modified arginyl-glycyl-aspartic acid (RGD), and 1,3-propanesultone (1,3-PS) in sequence to form RGD-Gd@Au-DNGs-PS (R-G@ADP, Figure 8a,b). The resultant colloidal-stable R-G@ADP possesses an average diameter of approximately 122 nm (Figure 8c) and possesses a high X-ray attenuation coefficient, excellent r_1_ relaxivity (Figure 8d, 9.13 mM^−1^ s^−1^), the desired protein resistance, and biocompatibility. Further, RGD enabled the targeted tumor penetration capability and enhanced magnetic resonance/computed tomography dual-modal imaging of pancreatic tumors [127].

In another study, ultrasound-enhanced cancer theranostics based on intelligent redox-responsive PAMAM G3 NGs loaded with Au NPs and toyocamycin (Au/Toy@G3 NGs) were showcased by the same group. The Au/Toy@G3 NGs have a diameter of about 193 nm and are colloidal-stabile, which dissociate to release the Au NPs and Toy (a chemotherapeutic drug) in the tumor microenvironment [128]. Through amplifying stress on the endoplasmic reticulum, Toy can promote the apoptosis of cancer cells and lead to immunogenic cell death in maturate dendritic cells. On the other hand, Au NPs can induce the conversion of M2-type tumor-associated macrophages into the antitumor M1-type to re-modulate the immunosuppressive tumor microenvironment. Coupled with antibody-mediated immune checkpoint blockade, effective pancreatic tumor chemo-immuno-therapy can be realized. Furthermore, their chemo-immuno-therapy effect on a mouse model can be ultrasound-enhanced due to the sonoporation-improved tumor permeability of the NGs. The Au/Toy@G3 NGs can also act as a computed tomography agent for tumor imaging. To enhance the delivery efficiency for long-term glaucoma therapy, researchers developed dendrimer hydrogel particles (DHPs) of various sizes, i.e., μDHP10 (9 μm), μDHP3 (3 μm), and nDHPs (nano-in-nano dendrimer hydrogel particles, ~200 nm), using an aza-Michael addition reaction, which was combined with the inverse emulsion method. Two first-line antiglaucoma drugs, timolol maleate and brimonidine tartrate, were loaded into the above dendrimer gel particles to study their delivery efficiency and efficacy. The conclusions elucidate that the nDHPs were superior to μDHP10 and μDHP3 concerning their biodegradability, biocompatibility, drug release, and corneal permeability. To be specific, the nDHPs were able to increase the corneal permeability 17-fold over the pristine drug solution and enabled zero-order sustained drug release. The nDHPs decreased the intraocular pressure in single-dose tests and a 7-day chronic daily dosing test in both Brown Norway rats and glaucoma mice [129]. Therefore, the nano-in-nano DHPs enable sustained release of and enhance the therapeutic efficacy of antiglaucoma drugs, which may facilitate enhanced clinical glaucoma treatment.

## 6. Conclusions and Outlook

PAMAM dendrimers can be used to engineer various polymer hydrogels. Hydrogels can store a variety of hydrophilic functional small molecules to structurally and functionally mimic the natural extracellular matrix and hence have been extensively studied for biomedical applications. This review briefly discusses recent advances in PAMAM-based hydrogels for biomedical applications, including drug delivery, tissue engineering, drug-free tumor therapy, and other related fields. In addition, the preparation methods for these hydrogels are briefly described. Therefore, this review aims to provide researchers with a valuable resource for understanding the current state of the art and facilitating further developments in functional hydrogel materials based on PAMAM dendrimers.

## 7. Perspectives

Although the design and application of PAMAM-engineered hydrogels have been extensively studied, there is still a long way to go for the real application of these hydrogels. Firstly, the availability of PAMAM dendrimers, especially high-generation PAMAM dendrimers, is limited. Even though PAMAM dendrimers G1-G10 have been made commercially available, their preparation requires expensive raw materials and specialized equipment, as well as complex preparation processes, which make the products inaccessible and restricts their clinical translational applications. However, with in-depth research and technological advancements, efforts are being made to find more economical and efficient preparation methods to reduce the cost of PAMAM and promote its application in various fields. For the currently available PAMAM dendrimers, future research could focus on tailoring the properties of PAMAM-engineered hydrogels to meet specific biomedical needs, such as enhanced biocompatibility, controlled drug release kinetics, and improved mechanical strength. This may be achieved by exploring new crosslinking strategies and surface functionalization or incorporating bioactive molecules. In addition, developing bioresponsive PAMAM-engineered hydrogels that can actively respond to physiological stimuli, such as pH, temperature, or enzymatic activity, enabling controlled drug release or targeted tissue engineering applications, could be another promising direction.

Secondly, investigating the potential of PAMAM-engineered hydrogels as multifunctional platforms for combined therapies could be an exciting avenue. This could involve exploring the synergistic effects between different therapeutic agents, such as drugs and genes. In addition to being used as drugs, dendrimers are widely used as carriers to deliver genetic materials (e.g., DNA, RNA) for targeted gene transfection. Furthermore, dendrimers can be loaded with functional materials, such as metal nanoparticles, fluorescent markers, etc., which are used in bio-imaging, tissue engineering, and drug delivery, etc. As a result, choosing certain hyperbranched polymers with high accessibility to structurally mimic dendrimers can promote research on the application of dendrimer-engineered hydrogels.

Thirdly, the molecular structure, molecular weight, number of active groups, hydrophilicity, and hydrophobicity of different-generation PAMAM dendrimers vary greatly. In general, high-generation dendrimers have complex structures, which give them unique performance characteristics. Therefore, in addition to the most frequently discussed, G3-G5, research on hydrogels functionalized based on higher-generation PAMAM dendrimers needs to be further advanced. This review will help researchers to design and develop more functional hydrogel materials based on PAMAM.

Lastly, reports indicate that linear poly(amidoamine)-engineered hydrogels exhibit excellent biodegradability, and their degradation products are completely noncytotoxic [130,131]. Therefore, PAMAM-dendrimer-engineered hydrogels may also exhibit biodegradability characteristics. Factors like the generation of the PAMAM dendrimers, the crosslinking density, the functional groups, and the environmental conditions should be systematically considered when tailoring their biodegradability.

## Figures and Tables

**Table 1:** A summary of hydrogel precursors, drug loading methods, and hydrogel formation methods.

Precursors	Drug	Drug Loading Method	Hydrogel Formation Method	Ref.
Polyethylene glycol bisazide, G4-functionalized dibenzocyclooctyne	5-fluorouracil	One-step strategy	Click chemistry reaction	[59]
Vinyl-sulfone-functionalized G5, thiolated polyethylene glycol (PEG)	Diflunisal	Two-step strategy		[60]
Vinyl-sulfone-functionalized G5, thiolated PEG	Silibinin, methotrexate, and camptothecin			[61]
G4-pentenoic acid conjugates, thiolated hyaluronic acid	Dexamethasone			[62]
G3, PEG diacrylate (PEG-DA)	Camptothecin (CPT)		Aza-Michael addition	[63]
G5, PEG-DA	Brimonidine tartrate	One-step strategy		[64]
G5, PEG-DA	5-fluorouracil			[65]
G5, PEG-DA	CPT	Two-step strategy	Aza-Michael addition and inverse microemulsion method	[66]
G4, glutaraldehyde	Ketoprofen		Schiff base reaction	[67]
G2, multi-armed PEG with N-succinimidyl ester end groups	Fluorescein isothiocyanate-dextran	One-step strategy	Amidation reaction	[68]
Tyramine-conjugated tetronic and p-hydroxyphenyl-acetic-acid-functionalized G3	Heparin		Enzymatic reaction	[69]
N-isopropy-lacrylamide, N,N′-methylenebis(acrylamide), and G6	Paracetamol	Direct binding to the hydrogel matrix	Radical polymerization	[70]
